# Close of Volume

**Published:** 1874-01

**Authors:** 


### The Bistoury.

ELMIRA, N. Y., JAN. 1874.

The BISTOURY is published Quarterly, upon the 1st of April, July, October and January, at Fifty Cents a year, in advance.

### CLOSE OF VOLUME.

With this number expires the old year, and Volume Nine of the BISTOURY. With the next number we commence the tenth year of the publication of our journal; it having risen from a diminutive and unpretending little sheet, to a first class health magazine, with a circulation larger than any medical journal in the country.

Many of our subscribers are sadly in arrears for the BISTOURY. We will be under lasting obligations to such, to at once make full payment of their indebtedness due for subscription, and order the journal discontinued, as it does not "pay" to carry delinquents from year to year, and we cannot afford to drop them from our list, and lose what they already owe.

In this number will be found the usual blank and envelope, in which to remit arrearages and subscription for the coming year.—We hope our readers will remit promptly, and strive to send a new name with their own. Extending the compliments of the season to our patrons all, we hope the BISTOURY will be as welcome and entertaining as of yore.

